# Transmission of the Herpes Simplex Virus in the Preclinical Phase of Disease Progression during Childbirth

**DOI:** 10.31662/jmaj.2022-0200

**Published:** 2023-03-13

**Authors:** Shunji Suzuki

**Affiliations:** 1 Department of Obstetrics and Gynecology, Japanese Red Cross Katsushika Maternity Hospital, Tokyo, Japan

**Keywords:** herpes simplex virus infection, birth-acquired herpes, vertical infection

## Abstract

Vertical transmission of the herpes simplex virus (HSV) in the preclinical phase of the disease progression is rare. Here, we present a case of perinatal-acquired herpes from an asymptomatic mother. Our findings suggest that clinicians should consider screening predisposed mothers for HSV as part of prenatal care to identify asymptomatic primary genital HSV infections.

## Introduction

Genital herpes simplex virus (HSV) infections are common sexually transmitted infections ^(1), (2)^, which are transmitted through intimate person-to-person contact. In the case of HSV-1, kissing or oral sex can spread the infection to another person, while HSV-2 can be contracted through vaginal, anal, or oral sex with someone who has the virus. The infections in women of reproductive age can carry the additional risk of vertical transmission to the neonate at the time of delivery ^(1), (2)^. Neonatal herpes infections develop after an incubation period of 2-10 days but are characterized by a lack of specific initial symptoms. Neonatal herpes infections have been reported to be devastating, with up to 50% mortality for disseminated HSV infections in the newborn ^(1), (2)^. In our previous study in 2016, the prevalence of genital HSV infections during pregnancy in Japan was one in 536 patients ^(3)^. Based on a nationwide epidemiological survey in Japan in 2020, the estimated number of neonates infected with HSV was 1 in 28,000 live births, and the ratio of HSV1:HSV 2 was almost 1:1 ^(4)^. Although the majority (85%) of neonatal HSV infections are acquired perinatally from the birth canal ^(2)^, most neonates with HSV disease are born to mothers without a history or symptoms of HSV infection ^(5)^. Only ~30% of mothers with genital herpes infections exhibit genital herpes findings ^(1), (2), (5)^. In addition, vertical transmission of HSV in a preclinical phase of the disease may be rare ^(1), (2), (5)^. However, in this study, we present a case of perinatally-acquired herpes from an asymptomatic mother.

## Case Report

**Figure 1.**
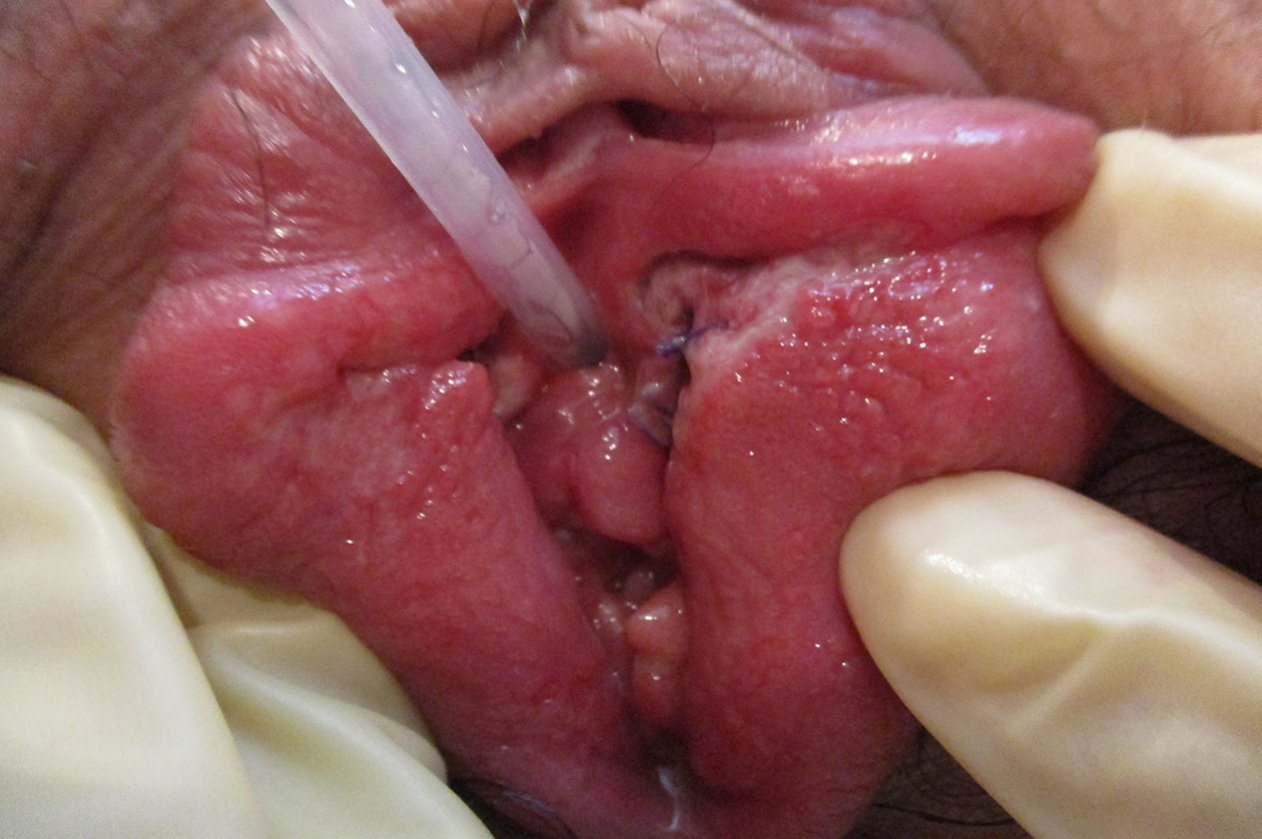
Blister and ulcer formation observed on the vulva.

**Figure 2.**
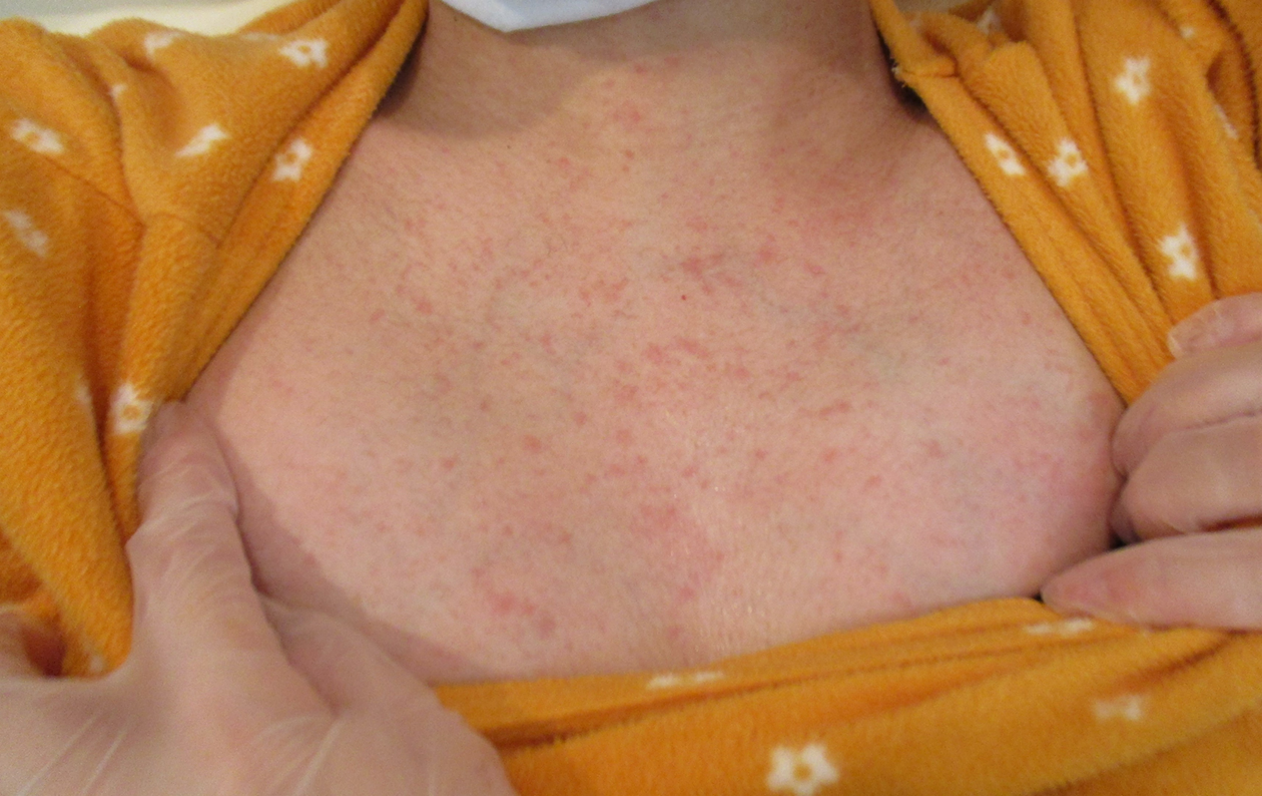
A rash on the precordium.

**Figure 3.**
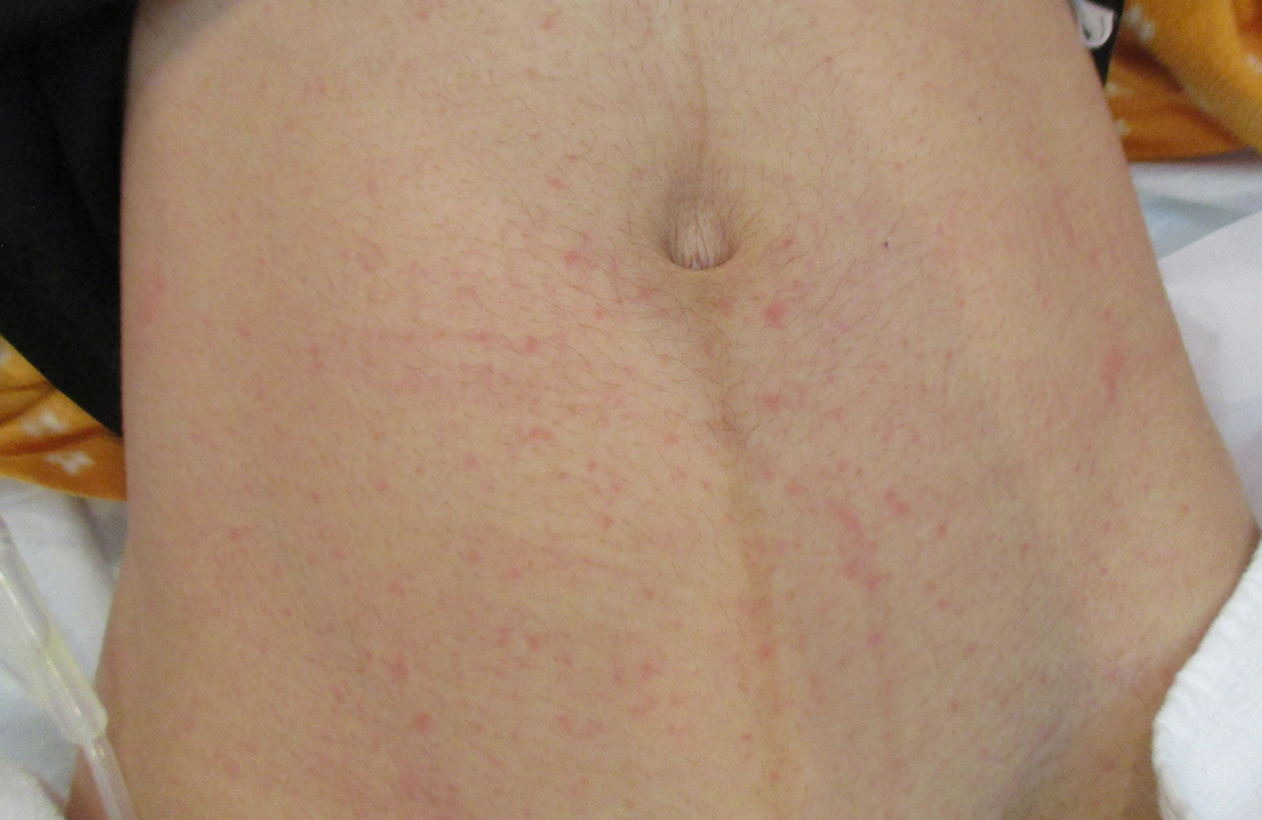
A rash on the abdomen.

A 21-year-old woman (gravida 1, para 0) spontaneously delivered a healthy male infant with Apgar scores of nine and 10 at 1 and 5 min, respectively, at 36 weeks and 4 days of gestation at our institute. She had no history of sexually transmitted diseases, including genital or labial HSV infections, or oral herpes. She has a history of having one partner, with whom she had intercourse for the first time in about a month with him a few days before the delivery, as she had been in the hospital for a long time due to premature labor at 34 weeks of gestation. Her pregnancy had progressed uneventfully. The arterial pH of the umbilical cord was 7.262. The mother and neonate had been healthy until the third day after delivery, and they were discharged the next day. However, the night of the mother’s discharge day, she revisited our institute, reporting concerns of fever of 40.0℃ and vulvar pain that developed after discharge. Blister and ulcer formation were observed on the vulva, indicating infection of the laceration suture (Figure 1). Her total white blood cell count was within the reference range (7,770/μL), but her C-reactive protein was elevated (5.52 mg/L). She had a rash on her precordium (Figure 2) and abdomen (Figure 3), indicating viral infections. HSV-2 infection was confirmed by a polymerase chain reaction assay of the vulva.

On the morning of the seventh day after birth, the newborn was feeding well and had no rash; however, in the afternoon of that day, he developed a fever of 38.0℃ and became dehydrated with hepatomegaly. His initial laboratory workup revealed an aspartate aminotransferase level of 3,763 IU/L and an alanine aminotransferase level of 1,083 IU/L. He was diagnosed with neonatal HSV infection, with HSV-2 detected in his serum and spinal fluid. The mother and neonate received long-term administration of acyclovir.

## Discussion

Here, we present a case of perinatal-acquired herpes from an asymptomatic mother. In the current case, we presumed her to be the primary HSV infection based on her sexual history obtained after the onset of the symptoms. However, because no antibodies against HSV were detected in her laboratory tests, which indicate seroconversion, and the vulvar lesion was not severe in the current case, we could not completely deny the possibility of the period before the clinical episode of reactivation of HSV infection. Nonetheless, the findings suggest that vertical transmission of HSV can occur before the first symptoms appear in the mother.

It is well-known that the initiation of long-term antiviral suppressive therapy at an early stage of HSV infection can significantly improve morbidity associated with the disease ^(6), (7)^. However, the initial action can sometimes be delayed due to the absence of typical findings of HSV infection in the mother and neonate because neonatal HSV infections are characterized by a lack of specific initial symptoms, developing after an incubation period of 2-10 days ^(8), (9), (10)^. In the current case, it was fortunate that the mother had symptoms of HSV infection 3 days before the newborn had a fever.

Mothers of infants who acquire neonatal herpes sometimes lack histories of clinically evident genital herpes, therefore, clinicians should recognize asymptomatic primary genital HSV infections ^(10)^. Based on the current rare case of vertical transmission of HSV from a young asymptomatic mother, HSV screening may be recommended prenatally in predisposed mothers such as adolescent women ^(3), (9)^.

Antiviral suppressive therapy at an early stage of neonatal HSV infection can lead to significant improvement in morbidity; however, such therapy is sometimes delayed due to the absence of typical findings of HSV infection in the mother and neonate. As this case highlights, asymptomatic mothers can give birth to neonates with HSV. The mortality rate for neonatal HSV infections in 2020 in Japan has been reported to be ~12% despite the widespread use of acyclovir, an effective antiviral medicine since the late 1980s ^(4)^; thus, clinicians should consider screening predisposed mothers for HSV as part of prenatal care to identify asymptomatic primary genital HSV infections, especially in young pregnant women, who have a particularly higher rate of the infection as compared to that of nonpregnant patients ^(3), (9)^.

## Article Information

### Conflicts of Interest

None

### Author Contributions

The author contributed to the study conception and design, performed data collection, made substantial contributions to the analyses and interpretation of the data, and wrote this manuscript.

### Informed Consent

The patient had agreed and signed informed consent to publish the case in an academic journal without exposing his identity.

### Approval by Institutional Review Board (IRB)

Not applicable
